# Supplementary material for: Developing a threat risk register based on the IUCN threat hierarchy for five tropical Important Plant Areas in Guinea
Source: PeerJ. 2025 Jul 14;13:e19629. doi: 10.7717/peerj.19629 (PMC12269778; doi:10.7717/peerj.19629)
Supplement: Supplemental Information 3 [file peerj-13-19629-s003.docx]

February 24, 2025

Dear Editors

We would like to explain the addition of Dr Martin Cheek as a co-author of the article.

Martin Cheek probably should have been a co-author from the start due to the contribution of data, though he did not impose this. In the revision of the paper, he has provided significant assistance with editing and expanding the discussion to provide clarity, this is reflected in the tracked changes documents. The co-authors would like to add him to reflect this and the use of the unpublished Kew data.

Kind regards

Charlotte Couch

Project Coordinator Tropical Important Plant Areas, Guinea

On behalf of all the authors.
